# Methyl 9-hy­droxy-15-methyl-2-oxo-11-(pyren-1-yl)-10-oxa-15-aza­tetra­cyclo­[7.6.0.0^1,12^.0^3,8^]penta­deca-3(8),4,6-triene-12-carboxyl­ate

**DOI:** 10.1107/S1600536813024951

**Published:** 2013-09-21

**Authors:** P. Sharmila, G. Jagadeesan, Rajesh Raju, Raghunathan Raghavachary, S. Aravindhan

**Affiliations:** a Department of Physics, Presidency College, Chennai 600 005, India; b Department of Organic Chemistry, University of Madras, Guindy Campus, Chennai 600 025, India

## Abstract

In the title compound, C_32_H_25_NO_5_, the furan and pyrrole rings each adopt an envelope conformation, the respective flap atoms being the C atom bearing the pyrene substituent and the CH_2_ atom adjacent to the N atom. The mol­ecular conformation is stabilized by an intra­molecular O—H⋯N hydrogen bond. In the crystal, C—H⋯O contacts link the mol­ecules, forming a two-dimensional network parallel to (001).

## Related literature

For the solid–state structures of pyrenes, see: Robertson & White (1947 ▶); Camerman & Trotter (1965 ▶); Allmann (1970 ▶); Hazell *et al.* (1972 ▶); Kai *et al.* (1978 ▶). For a related structure, see: Gruber *et al.* (2010 ▶). For the use of pyrenes in fluorescence sensors, see: Bren (2001 ▶).
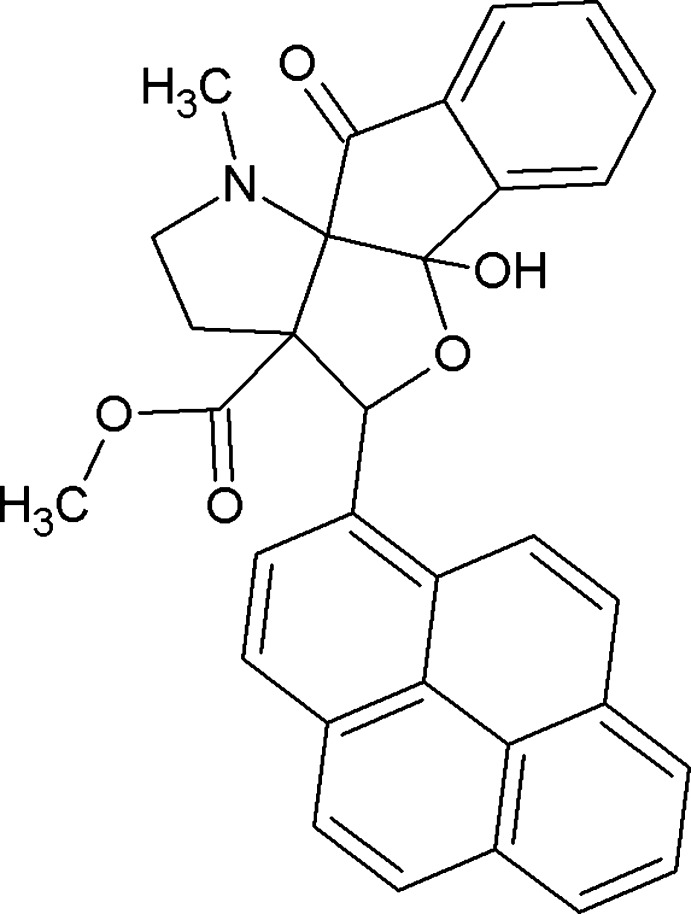


## Experimental

### 

#### Crystal data

C_32_H_25_NO_5_
*M* *_r_* = 503.53
Monoclinic, 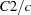

*a* = 31.6964 (6) Å
*b* = 11.0325 (2) Å
*c* = 14.1965 (3) Å
β = 96.503 (1)°
*V* = 4932.44 (17) Å^3^
*Z* = 8
Mo *K*α radiation
μ = 0.09 mm^−1^
*T* = 293 K
0.25 × 0.20 × 0.20 mm

#### Data collection

Bruker Kappa APEXII CCD diffractometer
Absorption correction: multi-scan (*SADABS*; Bruker, 2004 ▶) *T*_min_ = 0.979, *T*_max_ = 0.983
20953 measured reflections
4661 independent reflections
3280 reflections with *I* > 2σ(*I*)
*R*_int_ = 0.031

#### Refinement

*R*[*F* ^2^ > 2σ(*F* ^2^)] = 0.041
*wR*(*F* ^2^) = 0.114
*S* = 1.04
4661 reflections
347 parameters
H atoms treated by a mixture of independent and constrained refinement
Δρ_max_ = 0.15 e Å^−3^
Δρ_min_ = −0.18 e Å^−3^

### 

Data collection: *APEX2* (Bruker, 2004 ▶); cell refinement: *APEX2* and *SAINT* (Bruker, 2004 ▶); data reduction: *SAINT* and *XPREP* (Bruker, 2004 ▶); program(s) used to solve structure: *SHELXS97* (Sheldrick, 2008 ▶); program(s) used to refine structure: *SHELXL97* (Sheldrick, 2008 ▶); molecular graphics: *ORTEP-3 for Windows* (Farrugia, 2012 ▶); software used to prepare material for publication: *PLATON* (Spek, 2009 ▶).

## Supplementary Material

Crystal structure: contains datablock(s) I, 2R. DOI: 10.1107/S1600536813024951/bt6925sup1.cif

Structure factors: contains datablock(s) I. DOI: 10.1107/S1600536813024951/bt6925Isup2.hkl

Supplementary material file. DOI: 10.1107/S1600536813024951/bt6925Isup3.cml

Additional supplementary materials:  crystallographic information; 3D view; checkCIF report

## Figures and Tables

**Table 1:** Hydrogen-bond geometry (Å, °)

*D*—H⋯*A*	*D*—H	H⋯*A*	*D*⋯*A*	*D*—H⋯*A*
C27—H27⋯O3^i^	0.93	2.43	3.299 (2)	155
O2—H2⋯N	0.87 (2)	1.95 (2)	2.6217 (19)	133 (2)

Symmetry code: (i) 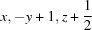.

**Fig. 1.**
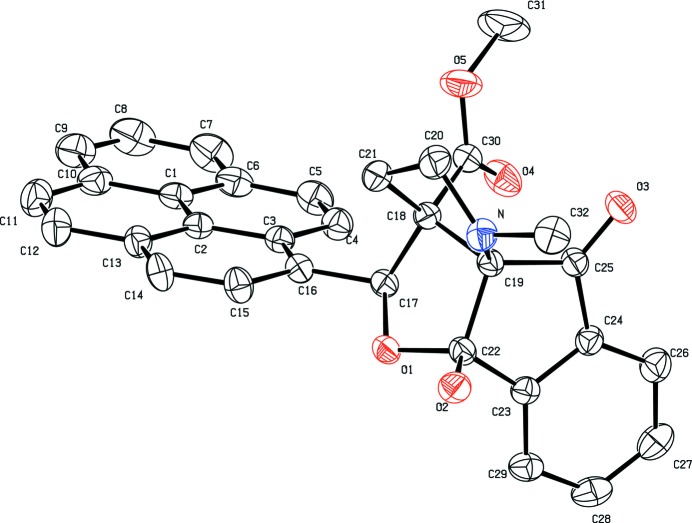
The molecular structure of the title compound, Displacement ellipsoids are drawn at the 30% probability level, H atoms have been omitted for clarity.

**Fig. 2.**
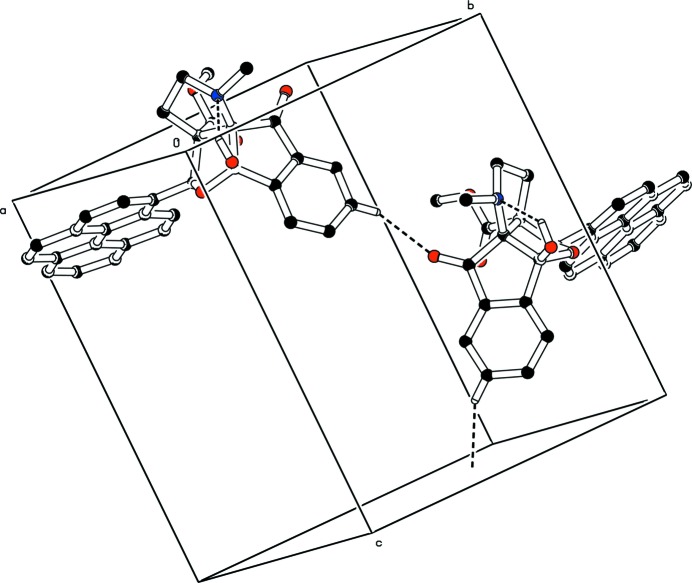
Crystal packing of the title compound, Hydrogen bonds are shown as dashed lines. For the sake of clarity, H atoms not involved in the interactions have been omitted.

## supplementary crystallographic information

### 1. Comment

Owing to their electronic, optical and geometric properties, mono-functionalized pyrenes, attachable to a receptor platform, are of special interest for the development of fluorescent sensors (Bren *et al.*, 2001).

The pyrene moiety alone shows no significant deviations of bond lengths and angles compared with those of the unsubstituted analogue (Robertson *et al.*, 1947; Camerman *et al.*, 1965; Allmann *et al.*, 1970; Hazell *et al.*, 1972; Kai *et al.*, 1978). The furan and pyrole rings adopt an envelope conformation. C17 and C20 are displaced by -0.2843 (2) Å and 0.2851 (2) Å, respectively, from the least-square planes formed by the remaining ring atoms. The dihedral angle between the furan and pyrole ring being 65.50 (6)°. The carboxylate group (C30/05/C31) is almost perpendicular to the furan ring with dihedral angle of 85.05 (1)°. The molecular conformation is stabilized by an intramolecular O—H···N hydrogen bond and the crystal packing is stabilized by intermolecular C—H···O contacts.

### 2. Experimental

To a reaction mixture of 2-((3*a*2, 4-dihydropyren-1-yl)(hydroxy)methyl)acrylate (1 mmol), ninhydrine (1.1 mmol) and sarcosine (1.1 mmol) was refluxed in methanol until completion of the reaction was evidenced by TLC analysis. After completion of the reaction the solvent was evaporated under reduced pressure. The crude reaction mixture was dissolved in dichloromethane and washed with water followed by brine solution. The organic layer was separated and dried over sodium sulfate, filtering and evaporation of the organic solvent under reduced pressure. The product was separated by column chromatography using hexane and ethyl acetate (3:7) as an eluent to give colorless solid. The product was dissolved in chloroform and heated for five minutes. The resulting solution was subjected to crystallization by slow evaporation of the solvent resulting in single crystals suitable for XRD studies.

### 3. Refinement

All the H atoms were positioned geometrically, with C–H = 0.93–0.97Å and constrained to ride on their parent atom, with *U*_iso_(H) =1.5*U*_eq_(C) for methyl H atoms and 1.2*U*_eq_(C) for other H atoms. The hydroxyl H atom was freely refined.

### Crystal data

**Table:**

C_32_H_25_NO_5_	*F*(000) = 2112
*M**_r_* = 503.53	*D*_x_ = 1.356 Mg m^−^^3^
Monoclinic, *C*2/*c*	Mo *K*α radiation, λ = 0.71073 Å
Hall symbol: -C 2yc	Cell parameters from 8834 reflections
*a* = 31.6964 (6) Å	θ = 2.1–31.2°
*b* = 11.0325 (2) Å	µ = 0.09 mm^−^^1^
*c* = 14.1965 (3) Å	*T* = 293 K
β = 96.503 (1)°	Block, colourless
*V* = 4932.44 (17) Å^3^	0.25 × 0.20 × 0.20 mm
*Z* = 8	

### Data collection

**Table:**

Bruker Kappa APEXII CCD diffractometer	4661 independent reflections
Radiation source: fine-focus sealed tube	3280 reflections with *I* > 2σ(*I*)
Graphite monochromator	*R*_int_ = 0.031
ω and φ scan	θ_max_ = 25.7°, θ_min_ = 2.0°
Absorption correction: multi-scan (*SADABS*; Bruker, 2004)	*h* = −38→37
*T*_min_ = 0.979, *T*_max_ = 0.983	*k* = −11→13
20953 measured reflections	*l* = −17→17

### Refinement

**Table:**

Refinement on *F*^2^	Primary atom site location: structure-invariant direct methods
Least-squares matrix: full	Secondary atom site location: difference Fourier map
*R*[*F*^2^ > 2σ(*F*^2^)] = 0.041	Hydrogen site location: inferred from neighbouring sites
*wR*(*F*^2^) = 0.114	H atoms treated by a mixture of independent and constrained refinement
*S* = 1.04	*w* = 1/[σ^2^(*F*_o_^2^) + (0.0547*P*)^2^ + 1.507*P*] where *P* = (*F*_o_^2^ + 2*F*_c_^2^)/3
4661 reflections	(Δ/σ)_max_ < 0.001
347 parameters	Δρ_max_ = 0.15 e Å^−^^3^
0 restraints	Δρ_min_ = −0.18 e Å^−^^3^

### Special details

**Table:**

Geometry. All e.s.d.'s (except the e.s.d. in the dihedral angle between two l.s. planes) are estimated using the full covariance matrix. The cell e.s.d.'s are taken into account individually in the estimation of e.s.d.'s in distances, angles and torsion angles; correlations between e.s.d.'s in cell parameters are only used when they are defined by crystal symmetry. An approximate (isotropic) treatment of cell e.s.d.'s is used for estimating e.s.d.'s involving l.s. planes.
Refinement. Refinement of *F*^2^ against ALL reflections. The weighted *R*-factor *wR* and goodness of fit *S* are based on *F*^2^, conventional *R*-factors *R* are based on *F*, with *F* set to zero for negative *F*^2^. The threshold expression of *F*^2^ > σ(*F*^2^) is used only for calculating *R*-factors(gt) *etc*. and is not relevant to the choice of reflections for refinement. *R*-factors based on *F*^2^ are statistically about twice as large as those based on *F*, and *R*- factors based on ALL data will be even larger.

### Fractional atomic coordinates and isotropic or equivalent isotropic displacement parameters (Å^2^)

**Table:**

	*x*	*y*	*z*	*U*_iso_*/*U*_eq_	
O1	0.06963 (4)	0.02788 (10)	0.10712 (8)	0.0468 (3)	
O3	0.08806 (4)	0.40608 (11)	−0.02173 (8)	0.0555 (3)	
O2	0.00524 (4)	0.11622 (12)	0.06940 (10)	0.0499 (3)	
N	0.03715 (5)	0.18310 (13)	−0.08500 (9)	0.0468 (4)	
C17	0.11197 (5)	0.04084 (15)	0.08207 (11)	0.0396 (4)	
H17	0.1286	0.0899	0.1304	0.048*	
C16	0.13183 (5)	−0.08345 (15)	0.07916 (11)	0.0412 (4)	
O4	0.16465 (4)	0.24317 (13)	0.03901 (10)	0.0647 (4)	
C1	0.23651 (5)	−0.24458 (17)	0.12311 (11)	0.0453 (4)	
C2	0.19242 (5)	−0.22187 (16)	0.09870 (11)	0.0408 (4)	
C3	0.17562 (5)	−0.10230 (15)	0.10426 (10)	0.0396 (4)	
O5	0.15453 (5)	0.19027 (15)	−0.11231 (10)	0.0828 (5)	
C23	0.05729 (5)	0.23187 (15)	0.16533 (11)	0.0387 (4)	
C25	0.07807 (5)	0.32732 (16)	0.03066 (11)	0.0402 (4)	
C10	0.25291 (6)	−0.36313 (19)	0.11528 (12)	0.0532 (5)	
C13	0.16523 (6)	−0.31982 (16)	0.06926 (12)	0.0452 (4)	
C15	0.10609 (6)	−0.18228 (16)	0.05246 (13)	0.0496 (5)	
H15	0.0771	−0.1702	0.0375	0.060*	
C22	0.04826 (5)	0.14005 (15)	0.08771 (11)	0.0396 (4)	
C18	0.10582 (5)	0.11345 (15)	−0.01307 (11)	0.0391 (4)	
C29	0.05082 (5)	0.22207 (18)	0.25959 (11)	0.0484 (5)	
H29	0.0407	0.1504	0.2832	0.058*	
C24	0.07323 (5)	0.33856 (16)	0.13185 (11)	0.0419 (4)	
C14	0.12230 (6)	−0.29750 (16)	0.04743 (13)	0.0510 (5)	
H14	0.1041	−0.3614	0.0290	0.061*	
C4	0.20465 (6)	−0.00815 (18)	0.13747 (12)	0.0491 (5)	
H4	0.1947	0.0708	0.1417	0.059*	
C5	0.24608 (6)	−0.0317 (2)	0.16270 (13)	0.0575 (5)	
H5	0.2638	0.0315	0.1855	0.069*	
C28	0.05960 (6)	0.3202 (2)	0.31740 (13)	0.0608 (6)	
H28	0.0545	0.3153	0.3805	0.073*	
C21	0.08922 (6)	0.03972 (17)	−0.10085 (12)	0.0496 (5)	
H21A	0.1123	0.0165	−0.1366	0.059*	
H21B	0.0747	−0.0329	−0.0831	0.059*	
C6	0.26386 (6)	−0.1491 (2)	0.15595 (13)	0.0541 (5)	
C20	0.05868 (6)	0.12456 (18)	−0.15795 (12)	0.0550 (5)	
H20A	0.0737	0.1832	−0.1926	0.066*	
H20B	0.0388	0.0801	−0.2022	0.066*	
C26	0.08287 (6)	0.43730 (18)	0.19105 (13)	0.0558 (5)	
H26	0.0937	0.5085	0.1682	0.067*	
C30	0.14512 (6)	0.18882 (16)	−0.02412 (13)	0.0461 (4)	
C12	0.18277 (7)	−0.43904 (19)	0.06394 (14)	0.0593 (5)	
H12	0.1650	−0.5037	0.0452	0.071*	
C11	0.22455 (7)	−0.4587 (2)	0.08562 (14)	0.0637 (6)	
H11	0.2351	−0.5368	0.0811	0.076*	
C19	0.06708 (5)	0.19699 (15)	0.00068 (10)	0.0375 (4)	
C32	0.00976 (7)	0.28537 (19)	−0.11593 (14)	0.0617 (5)	
H32A	−0.0029	0.3173	−0.0628	0.092*	
H32B	−0.0122	0.2587	−0.1636	0.092*	
H32C	0.0263	0.3474	−0.1418	0.092*	
C9	0.29646 (7)	−0.3813 (2)	0.13888 (15)	0.0695 (6)	
H9	0.3077	−0.4585	0.1332	0.083*	
C27	0.07588 (7)	0.4264 (2)	0.28456 (13)	0.0642 (6)	
H27	0.0821	0.4909	0.3259	0.077*	
C7	0.30703 (7)	−0.1735 (3)	0.17972 (16)	0.0736 (7)	
H7	0.3253	−0.1114	0.2023	0.088*	
C8	0.32282 (7)	−0.2884 (3)	0.17003 (17)	0.0783 (7)	
H8	0.3517	−0.3028	0.1848	0.094*	
C31	0.18922 (11)	0.2689 (3)	−0.1309 (2)	0.1279 (13)	
H31A	0.1935	0.2635	−0.1966	0.192*	
H31B	0.2147	0.2442	−0.0925	0.192*	
H31C	0.1825	0.3510	−0.1159	0.192*	
H2	0.0041 (7)	0.110 (2)	0.0079 (16)	0.081 (7)*	

### Atomic displacement parameters (Å^2^)

**Table:**

	*U*^11^	*U*^22^	*U*^33^	*U*^12^	*U*^13^	*U*^23^
O1	0.0466 (7)	0.0380 (7)	0.0591 (7)	0.0011 (5)	0.0194 (5)	0.0090 (6)
O3	0.0785 (9)	0.0419 (7)	0.0462 (7)	−0.0123 (7)	0.0079 (6)	0.0074 (6)
O2	0.0402 (7)	0.0526 (8)	0.0577 (8)	−0.0076 (6)	0.0101 (6)	−0.0048 (6)
N	0.0519 (9)	0.0460 (9)	0.0407 (7)	−0.0009 (7)	−0.0024 (6)	−0.0036 (7)
C17	0.0378 (9)	0.0375 (10)	0.0445 (9)	−0.0044 (8)	0.0080 (7)	0.0011 (7)
C16	0.0433 (10)	0.0377 (10)	0.0426 (9)	−0.0018 (8)	0.0047 (7)	0.0027 (7)
O4	0.0629 (9)	0.0629 (9)	0.0671 (9)	−0.0265 (8)	0.0021 (7)	0.0046 (7)
C1	0.0426 (11)	0.0574 (12)	0.0365 (8)	0.0016 (9)	0.0074 (7)	0.0088 (8)
C2	0.0437 (10)	0.0452 (10)	0.0337 (8)	0.0001 (8)	0.0060 (7)	0.0025 (7)
C3	0.0414 (10)	0.0424 (10)	0.0350 (8)	−0.0039 (8)	0.0050 (7)	0.0010 (7)
O5	0.1044 (13)	0.0818 (11)	0.0715 (9)	−0.0397 (9)	0.0505 (9)	−0.0140 (8)
C23	0.0334 (9)	0.0427 (10)	0.0402 (9)	0.0039 (8)	0.0045 (7)	0.0027 (7)
C25	0.0447 (10)	0.0365 (10)	0.0392 (8)	−0.0012 (8)	0.0035 (7)	0.0026 (7)
C10	0.0522 (12)	0.0634 (13)	0.0451 (10)	0.0141 (10)	0.0103 (8)	0.0123 (9)
C13	0.0527 (11)	0.0380 (10)	0.0440 (9)	0.0029 (9)	0.0013 (8)	0.0020 (8)
C15	0.0415 (10)	0.0410 (11)	0.0646 (11)	−0.0031 (9)	−0.0018 (8)	0.0034 (9)
C22	0.0370 (10)	0.0358 (10)	0.0468 (9)	−0.0008 (7)	0.0085 (7)	0.0041 (7)
C18	0.0414 (10)	0.0371 (10)	0.0397 (8)	−0.0041 (8)	0.0087 (7)	−0.0025 (7)
C29	0.0442 (10)	0.0612 (12)	0.0399 (9)	0.0072 (9)	0.0046 (7)	0.0085 (9)
C24	0.0469 (10)	0.0391 (10)	0.0393 (8)	0.0012 (8)	0.0035 (7)	−0.0036 (8)
C14	0.0511 (12)	0.0347 (10)	0.0642 (11)	−0.0068 (8)	−0.0061 (9)	0.0029 (9)
C4	0.0476 (11)	0.0477 (11)	0.0517 (10)	−0.0047 (9)	0.0039 (8)	−0.0019 (8)
C5	0.0487 (12)	0.0633 (14)	0.0594 (11)	−0.0167 (10)	0.0009 (9)	0.0015 (10)
C28	0.0600 (13)	0.0856 (16)	0.0369 (9)	0.0064 (12)	0.0066 (8)	−0.0028 (10)
C21	0.0592 (12)	0.0443 (11)	0.0462 (9)	−0.0039 (9)	0.0103 (8)	−0.0077 (8)
C6	0.0433 (11)	0.0684 (14)	0.0510 (10)	−0.0006 (10)	0.0075 (8)	0.0128 (10)
C20	0.0718 (13)	0.0519 (12)	0.0400 (9)	−0.0019 (10)	0.0009 (9)	−0.0079 (8)
C26	0.0668 (13)	0.0486 (12)	0.0515 (11)	−0.0036 (10)	0.0039 (9)	−0.0078 (9)
C30	0.0496 (11)	0.0405 (10)	0.0505 (10)	−0.0034 (9)	0.0153 (9)	0.0007 (8)
C12	0.0681 (14)	0.0445 (12)	0.0634 (12)	0.0053 (10)	−0.0009 (10)	−0.0007 (9)
C11	0.0729 (15)	0.0534 (13)	0.0647 (12)	0.0182 (12)	0.0071 (11)	0.0018 (10)
C19	0.0424 (10)	0.0343 (9)	0.0358 (8)	−0.0038 (7)	0.0040 (7)	−0.0005 (7)
C32	0.0629 (13)	0.0666 (14)	0.0523 (11)	0.0053 (11)	−0.0078 (9)	−0.0007 (10)
C9	0.0585 (14)	0.0812 (17)	0.0708 (13)	0.0222 (13)	0.0164 (11)	0.0211 (12)
C27	0.0731 (14)	0.0703 (15)	0.0484 (11)	0.0027 (12)	0.0027 (10)	−0.0226 (10)
C7	0.0442 (13)	0.0909 (19)	0.0843 (15)	−0.0056 (12)	0.0016 (11)	0.0195 (13)
C8	0.0431 (13)	0.103 (2)	0.0890 (16)	0.0141 (14)	0.0087 (11)	0.0327 (15)
C31	0.152 (3)	0.123 (3)	0.126 (2)	−0.074 (2)	0.091 (2)	−0.015 (2)

### Geometric parameters (Å, º)

**Table:**

O1—C22	1.423 (2)	C18—C21	1.531 (2)
O1—C17	1.4340 (18)	C18—C19	1.565 (2)
O3—C25	1.2090 (19)	C29—C28	1.368 (3)
O2—C22	1.384 (2)	C29—H29	0.9300
O2—H2	0.87 (2)	C24—C26	1.389 (2)
N—C20	1.455 (2)	C14—H14	0.9300
N—C32	1.460 (2)	C4—C5	1.347 (3)
N—C19	1.464 (2)	C4—H4	0.9300
C17—C16	1.511 (2)	C5—C6	1.420 (3)
C17—C18	1.563 (2)	C5—H5	0.9300
C17—H17	0.9800	C28—C27	1.382 (3)
C16—C15	1.389 (2)	C28—H28	0.9300
C16—C3	1.409 (2)	C21—C20	1.514 (3)
O4—C30	1.192 (2)	C21—H21A	0.9700
C1—C6	1.410 (3)	C21—H21B	0.9700
C1—C10	1.416 (3)	C6—C7	1.398 (3)
C1—C2	1.423 (2)	C20—H20A	0.9700
C2—C13	1.415 (2)	C20—H20B	0.9700
C2—C3	1.428 (2)	C26—C27	1.376 (3)
C3—C4	1.432 (2)	C26—H26	0.9300
O5—C30	1.319 (2)	C12—C11	1.343 (3)
O5—C31	1.448 (3)	C12—H12	0.9300
C23—C29	1.381 (2)	C11—H11	0.9300
C23—C24	1.386 (2)	C32—H32A	0.9600
C23—C22	1.500 (2)	C32—H32B	0.9600
C25—C24	1.467 (2)	C32—H32C	0.9600
C25—C19	1.529 (2)	C9—C8	1.364 (3)
C10—C9	1.398 (3)	C9—H9	0.9300
C10—C11	1.418 (3)	C27—H27	0.9300
C13—C14	1.383 (2)	C7—C8	1.375 (3)
C13—C12	1.433 (3)	C7—H7	0.9300
C15—C14	1.376 (3)	C8—H8	0.9300
C15—H15	0.9300	C31—H31A	0.9600
C22—C19	1.563 (2)	C31—H31B	0.9600
C18—C30	1.521 (2)	C31—H31C	0.9600
			
C22—O1—C17	107.67 (12)	C4—C5—C6	122.74 (19)
C22—O2—H2	97.4 (15)	C4—C5—H5	118.6
C20—N—C32	116.09 (14)	C6—C5—H5	118.6
C20—N—C19	109.02 (14)	C29—C28—C27	121.79 (17)
C32—N—C19	118.45 (14)	C29—C28—H28	119.1
O1—C17—C16	108.71 (13)	C27—C28—H28	119.1
O1—C17—C18	103.69 (12)	C20—C21—C18	104.32 (14)
C16—C17—C18	116.93 (13)	C20—C21—H21A	110.9
O1—C17—H17	109.1	C18—C21—H21A	110.9
C16—C17—H17	109.1	C20—C21—H21B	110.9
C18—C17—H17	109.1	C18—C21—H21B	110.9
C15—C16—C3	118.99 (16)	H21A—C21—H21B	108.9
C15—C16—C17	119.09 (15)	C7—C6—C1	119.0 (2)
C3—C16—C17	121.91 (15)	C7—C6—C5	123.0 (2)
C6—C1—C10	119.89 (17)	C1—C6—C5	118.03 (17)
C6—C1—C2	119.93 (17)	N—C20—C21	102.50 (13)
C10—C1—C2	120.17 (17)	N—C20—H20A	111.3
C13—C2—C1	119.05 (16)	C21—C20—H20A	111.3
C13—C2—C3	120.21 (15)	N—C20—H20B	111.3
C1—C2—C3	120.74 (16)	C21—C20—H20B	111.3
C16—C3—C2	119.03 (15)	H20A—C20—H20B	109.2
C16—C3—C4	123.66 (16)	C27—C26—C24	117.84 (19)
C2—C3—C4	117.30 (15)	C27—C26—H26	121.1
C30—O5—C31	115.97 (18)	C24—C26—H26	121.1
C29—C23—C24	119.91 (16)	O4—C30—O5	123.62 (17)
C29—C23—C22	128.47 (16)	O4—C30—C18	124.01 (15)
C24—C23—C22	111.60 (13)	O5—C30—C18	112.35 (16)
O3—C25—C24	127.25 (16)	C11—C12—C13	120.96 (19)
O3—C25—C19	124.89 (14)	C11—C12—H12	119.5
C24—C25—C19	107.84 (13)	C13—C12—H12	119.5
C9—C10—C1	118.4 (2)	C12—C11—C10	121.56 (19)
C9—C10—C11	122.7 (2)	C12—C11—H11	119.2
C1—C10—C11	118.93 (17)	C10—C11—H11	119.2
C14—C13—C2	118.74 (16)	N—C19—C25	115.83 (13)
C14—C13—C12	121.95 (17)	N—C19—C22	110.19 (13)
C2—C13—C12	119.31 (17)	C25—C19—C22	104.67 (12)
C14—C15—C16	121.89 (17)	N—C19—C18	106.18 (12)
C14—C15—H15	119.1	C25—C19—C18	115.56 (13)
C16—C15—H15	119.1	C22—C19—C18	103.71 (12)
O2—C22—O1	107.96 (13)	N—C32—H32A	109.5
O2—C22—C23	111.60 (13)	N—C32—H32B	109.5
O1—C22—C23	113.46 (13)	H32A—C32—H32B	109.5
O2—C22—C19	112.58 (13)	N—C32—H32C	109.5
O1—C22—C19	106.66 (12)	H32A—C32—H32C	109.5
C23—C22—C19	104.54 (13)	H32B—C32—H32C	109.5
C30—C18—C21	114.63 (14)	C8—C9—C10	121.4 (2)
C30—C18—C17	110.23 (13)	C8—C9—H9	119.3
C21—C18—C17	115.39 (14)	C10—C9—H9	119.3
C30—C18—C19	110.75 (13)	C26—C27—C28	120.44 (18)
C21—C18—C19	102.26 (13)	C26—C27—H27	119.8
C17—C18—C19	102.52 (12)	C28—C27—H27	119.8
C28—C29—C23	118.49 (18)	C8—C7—C6	120.7 (2)
C28—C29—H29	120.8	C8—C7—H7	119.6
C23—C29—H29	120.8	C6—C7—H7	119.6
C23—C24—C26	121.50 (15)	C9—C8—C7	120.6 (2)
C23—C24—C25	110.28 (15)	C9—C8—H8	119.7
C26—C24—C25	128.21 (16)	C7—C8—H8	119.7
C15—C14—C13	121.10 (17)	O5—C31—H31A	109.5
C15—C14—H14	119.5	O5—C31—H31B	109.5
C13—C14—H14	119.5	H31A—C31—H31B	109.5
C5—C4—C3	121.22 (18)	O5—C31—H31C	109.5
C5—C4—H4	119.4	H31A—C31—H31C	109.5
C3—C4—H4	119.4	H31B—C31—H31C	109.5
			
C22—O1—C17—C16	−166.42 (13)	C10—C1—C6—C7	0.4 (3)
C22—O1—C17—C18	−41.34 (15)	C2—C1—C6—C7	179.86 (16)
O1—C17—C16—C15	31.7 (2)	C10—C1—C6—C5	180.00 (16)
C18—C17—C16—C15	−85.17 (19)	C2—C1—C6—C5	−0.6 (2)
O1—C17—C16—C3	−147.21 (14)	C4—C5—C6—C7	178.29 (18)
C18—C17—C16—C3	95.89 (18)	C4—C5—C6—C1	−1.3 (3)
C6—C1—C2—C13	−177.65 (15)	C32—N—C20—C21	170.80 (15)
C10—C1—C2—C13	1.8 (2)	C19—N—C20—C21	33.93 (18)
C6—C1—C2—C3	2.0 (2)	C18—C21—C20—N	−40.11 (18)
C10—C1—C2—C3	−178.61 (14)	C23—C24—C26—C27	−0.4 (3)
C15—C16—C3—C2	1.9 (2)	C25—C24—C26—C27	−179.51 (18)
C17—C16—C3—C2	−179.16 (14)	C31—O5—C30—O4	−3.6 (3)
C15—C16—C3—C4	−176.69 (16)	C31—O5—C30—C18	174.6 (2)
C17—C16—C3—C4	2.3 (2)	C21—C18—C30—O4	−173.00 (18)
C13—C2—C3—C16	−0.6 (2)	C17—C18—C30—O4	−40.8 (2)
C1—C2—C3—C16	179.76 (14)	C19—C18—C30—O4	71.9 (2)
C13—C2—C3—C4	178.05 (14)	C21—C18—C30—O5	8.9 (2)
C1—C2—C3—C4	−1.6 (2)	C17—C18—C30—O5	141.05 (16)
C6—C1—C10—C9	−1.3 (2)	C19—C18—C30—O5	−106.19 (17)
C2—C1—C10—C9	179.25 (15)	C14—C13—C12—C11	−179.88 (18)
C6—C1—C10—C11	177.55 (16)	C2—C13—C12—C11	−0.6 (3)
C2—C1—C10—C11	−1.9 (2)	C13—C12—C11—C10	0.5 (3)
C1—C2—C13—C14	178.75 (15)	C9—C10—C11—C12	179.54 (18)
C3—C2—C13—C14	−0.9 (2)	C1—C10—C11—C12	0.7 (3)
C1—C2—C13—C12	−0.5 (2)	C20—N—C19—C25	115.29 (16)
C3—C2—C13—C12	179.85 (15)	C32—N—C19—C25	−20.4 (2)
C3—C16—C15—C14	−1.7 (3)	C20—N—C19—C22	−126.17 (15)
C17—C16—C15—C14	179.29 (16)	C32—N—C19—C22	98.12 (17)
C17—O1—C22—O2	153.13 (13)	C20—N—C19—C18	−14.48 (17)
C17—O1—C22—C23	−82.64 (15)	C32—N—C19—C18	−150.18 (15)
C17—O1—C22—C19	31.92 (16)	O3—C25—C19—N	−47.9 (2)
C29—C23—C22—O2	64.5 (2)	C24—C25—C19—N	130.74 (15)
C24—C23—C22—O2	−113.96 (16)	O3—C25—C19—C22	−169.45 (16)
C29—C23—C22—O1	−57.7 (2)	C24—C25—C19—C22	9.20 (17)
C24—C23—C22—O1	123.81 (15)	O3—C25—C19—C18	77.2 (2)
C29—C23—C22—C19	−173.51 (16)	C24—C25—C19—C18	−104.17 (15)
C24—C23—C22—C19	8.00 (18)	O2—C22—C19—N	−14.03 (19)
O1—C17—C18—C30	151.21 (13)	O1—C22—C19—N	104.20 (15)
C16—C17—C18—C30	−89.18 (17)	C23—C22—C19—N	−135.34 (14)
O1—C17—C18—C21	−76.99 (16)	O2—C22—C19—C25	111.15 (15)
C16—C17—C18—C21	42.6 (2)	O1—C22—C19—C25	−130.62 (13)
O1—C17—C18—C19	33.26 (15)	C23—C22—C19—C25	−10.16 (16)
C16—C17—C18—C19	152.87 (14)	O2—C22—C19—C18	−127.32 (14)
C24—C23—C29—C28	1.4 (3)	O1—C22—C19—C18	−9.09 (16)
C22—C23—C29—C28	−176.98 (17)	C23—C22—C19—C18	111.37 (13)
C29—C23—C24—C26	−0.2 (3)	C30—C18—C19—N	111.87 (15)
C22—C23—C24—C26	178.41 (16)	C21—C18—C19—N	−10.71 (16)
C29—C23—C24—C25	179.07 (15)	C17—C18—C19—N	−130.55 (13)
C22—C23—C24—C25	−2.3 (2)	C30—C18—C19—C25	−18.05 (18)
O3—C25—C24—C23	173.97 (17)	C21—C18—C19—C25	−140.62 (14)
C19—C25—C24—C23	−4.64 (19)	C17—C18—C19—C25	99.53 (14)
O3—C25—C24—C26	−6.8 (3)	C30—C18—C19—C22	−131.98 (14)
C19—C25—C24—C26	174.59 (18)	C21—C18—C19—C22	105.45 (14)
C16—C15—C14—C13	0.2 (3)	C17—C18—C19—C22	−14.40 (15)
C2—C13—C14—C15	1.1 (3)	C1—C10—C9—C8	0.9 (3)
C12—C13—C14—C15	−179.64 (17)	C11—C10—C9—C8	−177.93 (19)
C16—C3—C4—C5	178.38 (16)	C24—C26—C27—C28	−0.3 (3)
C2—C3—C4—C5	−0.2 (2)	C29—C28—C27—C26	1.5 (3)
C3—C4—C5—C6	1.7 (3)	C1—C6—C7—C8	0.9 (3)
C23—C29—C28—C27	−2.0 (3)	C5—C6—C7—C8	−178.6 (2)
C30—C18—C21—C20	−89.05 (18)	C10—C9—C8—C7	0.5 (3)
C17—C18—C21—C20	141.26 (15)	C6—C7—C8—C9	−1.4 (3)
C19—C18—C21—C20	30.85 (16)		

### Hydrogen-bond geometry (Å, º)

**Table:**

*D*—H···*A*	*D*—H	H···*A*	*D*···*A*	*D*—H···*A*
C27—H27···O3^i^	0.93	2.43	3.299 (2)	155
O2—H2···N	0.87 (2)	1.95 (2)	2.6217 (19)	133 (2)

Symmetry code: (i) *x*, −*y*+1, *z*+1/2.
